# Incidence and management of retrobulbar hemorrhage after blowout fracture repair

**DOI:** 10.1186/s12886-021-01943-1

**Published:** 2021-04-22

**Authors:** Jae Hwi Park, Inhye Kim, Jun Hyuk Son

**Affiliations:** 1 Department of Oculoplasty, Nune Eye Hospital, Daegu, South Korea; 2 Department of Ophthalmology, Yeungnam University Medical Center, 317-1, Daemyung dong, Nam-Gu, Daegu, 705-035 South Korea

**Keywords:** Retrobulbar hemorrhage, Orbital wall fracture, Postoperative blindness, Orbital compartment syndrome

## Abstract

**Background:**

Retrobulbar hemorrhage (RBH) is a rare complication after orbital surgery but associated with ocular complications including blindness. The aim of this study was to identify clinical characteristics of patients with RBH requiring emergent orbital decompression after blowout fracture repair.

**Method:**

A retrospective review of 426 blowout fracture patients at a tertiary oculoplastic clinic provided data regarding demographics, physical examination findings, and computed tomography (CT) images. Extraocular motility had been recorded in patient charts on a scale from 0 to − 4. Patients requiring emergent orbital decompression due to RBH after surgery (RBH group) were compared with those who did not (Control group), using the Mann-Whitney U-test. Incidences of RBH according to primary or secondary surgery were also investigated, using Fisher’s exact test.

**Result:**

Five (1.2%) of the 426 patients who underwent blowout fracture repair developed RBH requiring emergent intervention. All RBH patients fully recovered after the decompression procedure or conservative treatment. Number of days to surgery was significantly longer in the RBH group (97.0 ± 80.1) than in the Control group (29.0 ± 253.0) (*p* = 0.05). Preoperative enophthalmos was also significantly greater in the RBH group (RBH vs. Control group, 3.6 ± 1.7 mm versus 1.2 ± 1.3 mm (*p* = 0.003)). The incidence of RBH was significantly higher in patients that underwent secondary surgery (odds ratio = 92.9 [95% confidence interval, 11.16–773.23], *p* = 0.001).

**Conclusions:**

Surgeons should pay more attention to hemostasis and postoperative care in patients with a large preoperative enophthalmic eye, when time from injury to surgery is long and in revision cases. When RBH occurs, time to intervention and surgical decompression is critical for visual recovery and preventing blindness.

**Trial registration:**

The institutional review board of the Yeungnam University Medical Center approved this study (YUMC 2018-11-010), which was conducted in accord with the Declaration of Helsinki.

## Introduction

Retrobulbar hemorrhage (RBH) is a rare postoperative complication feared by ophthalmologists, because it can lead to blindness if not appropriately managed. Few studies have addressed the incidence of RBH after ophthalmic surgery, which is considered to have an extremely low incidence. Several case reports have been issued on the occurrence of postoperative RBH after oculoplastic surgery [1–5], and incidence rates of 0.055 to 3.2% have been reported, depending on type of surgery [6, 7].

Orbital fracture is a common consequence of facial trauma, and orbital fracture repair is a common orbital surgery performed in the ophthalmic plastic surgery departments of tertiary hospitals. Although RBH is a potentially fatal complication that can occur after such surgery, research on its incidence and risk factors is limited.

The study was conducted on the incidence, treatment outcomes, and clinical features of RBH in patients after blowout fracture repair. The purpose of this study was to identify the incidence and risk factors of RBH after isolated blowout fracture correction. The study was performed on a relatively large number of patients treated at a single tertiary hospital by a single operator using the same technique to improve understanding of the risk factors and management of RBH.

## Methods

The institutional review board of the Yeungnam university medical center approved this study (YUMC 2018–11-010), which was conducted in accord with the Declaration of Helsinki. Patients treated between January 2008 and December 2016 were eligible for inclusion.

The operation database of the Yeungnam university medical center was searched for orbital wall fracture repair operative codes to identify patients who had undergone blowout fracture repair. Medical records were screened by chart review to identify patients that had experienced solely blowout fracture repair; patients that underwent blowout fracture repair combined another repair of facial bone were excluded. All operations were performed by one surgeon (JH Son) at a single center.

Data collection included patient demographics, mechanism of injury, fracture location, and time elapsed between injury and surgical repair. Preoperative and postoperative clinical findings collected included visual acuity, presence of diplopia, eye motility, and exophthalmometry using a Hertel exophthalmometer. Need for muscle surgery or prisms and postoperative complications were also recorded. All patients taking anticoagulants prior to surgery were checked for medications, and then, after appropriate counselling, medications were discontinued for as long as necessary. In this study, we defined RBH as a posterior orbital hemorrhagic condition requiring additional intervention rather than routine treatment, which involved intravenous antibiotics and steroids (dexamethasone 5 mg) after surgery. The routine patients were discharged1–2 days after surgery, and prescribed oral antibiotics, steroids (prednisolone 20 mg), analgesics, and steroid eye drops for 1 week.

Fracture sizes were estimated by orbital computed tomography (CT) at time of diagnosis. To determine the apparent size of the fracture, we measured maximal bony defect diameters on coronal CT images.

Eye motility was assessed in supraduction, infraduction, adduction, and abduction. Duction limitation was graded using the following scale;0 (no limitation), − 1 (duction 30°–45°), − 2 (15°–30°), − 3 (duction< 15°), and − 4 (no movement) [8, 9]. Total duction limitation was calculated by summing ductions in the 4 positions of gaze [10].

Orbital fracture repair was performed using the transconjunctival approach. If needed, a lateral canthotomy incision and cantholysis of the inferior crus of the lateral canthal ligament was performed. The incision was extended to include a retrocaruncular incision when repair involved both the orbital floor and medial orbital wall. Complete reduction of orbital contents from fracture sites was performed prior to implant placement. Porous polyethylene (Medpor, Stryker, Kalamazoo, Michigan,USA) implants were used. In each case, the conjunctival incision was closed using an 8–0 Vicryl suture (Ethicon Inc., New Jersey, NY, USA). All patients were given a short course of oral steroids and oral antibiotics, and were discharged 1 or 2 days after surgery.

**Fig. 1:**
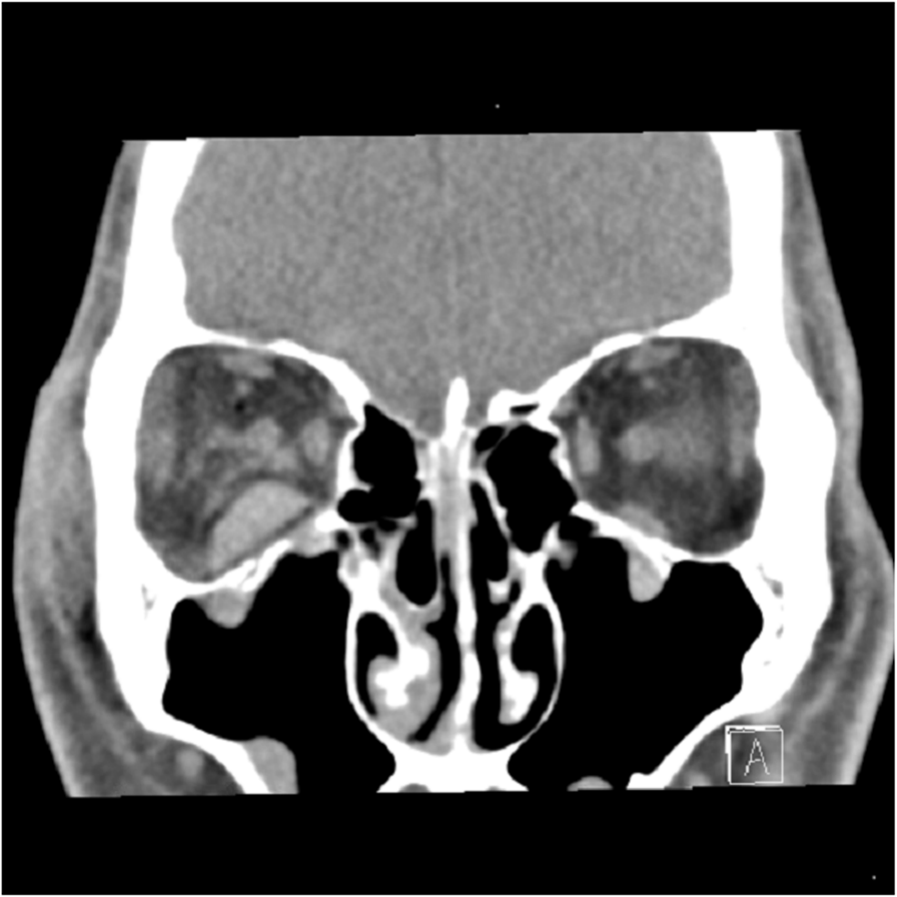
Computed tomographic image demonstrating retrobulbar hemorrhage at the inferior orbital rim after orbital blow-out fracture repair

Postoperative vision and pupil reaction checks were conducted in a general ward after awakening (range, 1 h ~ 4 h after surgery) by the operator. Vision checks were continued by ophthalmology residents on the morning of the following day. Diagnosis of RBH was established based on symptoms such as periocular pain and pressure, proptosis, eyelid discoloration, mydriasis, visual impairment, and diplopia. As soon as RBH was recognized, simple needle aspiration was attempted, and if the amount of orbital decompression was insufficient, a CT scan was performed as soon as possible (Fig. 1). After determining the location and amount of hematoma, lateral canthotomy and cantholysis were performed immediately. If CT was not possible within a short period of time, surgical procedures were immediately performed to avoid time delays. Intravenous high-dose steroid therapy was administered to patients when RBH was confirmed and best corrected visual acuity remained above 20/200.

Descriptive statistics were used to analyze demographics, incidence rates, and follow-up durations. Patients that developed RBH or did not develop RBH were compared. Continuous variables were analyzed using the Mann-Whitney U test and categorical variables using Fisher’s exact test. The analysis was performed using PASW Statistics Ver. 18 (IBM Software, USA).

## Results

Four hundred and twenty-six patients underwent isolated blowout fracture repair at Yeungnam university medical center from January 2008 through December 2016. The clinical characteristics of the 426 patients that underwent blowout fracture repair during the 9-year review period are summarized in Table 1.

**Table 1:** Patient Demographics

	Total
**Number of patients**	426
**Gender**	
**Male**	324
**Female**	102
**Age**	
**Average**	32.8 ± 15.2
**Range**	6 ~ 78
**Mechanism of injury**	
**Assault**	130
**Traffic accident**	34
**Sports**	87
**Slip/Fell down**	87
**Other accident**	88
**Location of fracture**	
**Inferior**	148
**Medial**	155
**Inferomedial**	123
**Size of fracture (mm)**	
**Average**	11.33 ± 6.00
**Range**	0.84 ~ 69.64
**Preoperative enophthalmos**	1.24 ± 1.36
**Days to surgery**	
**Average**	22.06 ± 35.98
**Range**	0 ~ 276
**Follow-up (months)**	5.23 ± 8.42

Times from injury to surgery ranged from 0 days to 276 days with a mean of 22 days (±36 days). Average fracture size was 11.33 mm (±6 mm). Among the 426 study subjects, 148 had an inferior orbital wall fracture, 155 had a medial orbital wall fracture, and 123 had an inferomedial orbital wall fracture.

Twenty-two complications were reported, of which 5 were RBH (1.2%). Postoperative complications are summarized in Table 2.

**Table 2:** Postoperative complications

	Number of patients (%)
**RBH**	5 (1.2%)
**Infection**	1 (0.2%)
**Implant exposure**	1 (0.2%)
**CRAO**^a^	1 (0.2%)
**Conjunctival granuloma**	6 (1.4%)
**Epiphora**	8 (1.9%)

^a^*CRAO* Central retinal artery occlusion

In no case did diplopia worsen after surgery. A summary of surgical and postoperative courses is provided in Table 3. Among the five RBH patients (the RBH group), lateral canthotomy and cantholysis were performed in 3, and sufficient decompression was achieved in one by needle aspiration only. The other patient received only high dose steroid treatment during hospitalization. Vision returned to normal in all 5 patients.

**Table 3:** Overview of patients with RBH after blowout fracture repair

Patient No.	Age	Gender	Days to surgery	Primary or Secondary	Preop.^a^ EOM^b^ limitation	Preop. enophthalmos (mm)	Fracture site	Fracture size (mm)	Point of occurrence	VA^c^ at diagnosis of RBH	Postop.^e^ pupil reaction	Intervention	Preop. VA	Postop. VA
1	33	M	163	Primary	−3	5	Inferomedial	12.23	POD^d^#0	HM^f^	RAPD	Canthotomy	20/20	20/20
2	40	M	148	Secondary	−12	4	Inferomedial	14.41	POD#4	HM	RAPD	Canthotomy	20/20	20/20
3	30	M	4	Primary	−0.5	3	Inferior	10.02	POD#12	20/200	RAPD	Aspiration	20/20	20/20
4	21	M	155	Secondary	0	5	medial	14.77	POD#4	60/200	RAPD	High dose steroid	20/20	20/20
5	34	M	15	Primary	0	1	Inferior	5.48	POD#0	FC^g^ 10 cm	RAPD	Canthotomy	20/20	20/20

^a^ *Preop.* preoperative, ^b^ *EOM* extraocular movement, ^c^ *VA* visual acuity, ^d^ *POD* postoperative day, ^e^ *Postop.* postoperative, ^f^ *HM* hand motion, ^g^ *FC* finger count

No significant differences were observed between the RBH and Control groups with respect to gender, age, mechanism of injury, location of fracture, size of fracture, or follow-up duration. However, significant intergroup differences were observed for preoperative enophthalmos (RBH vs. Control group, 3.6 ± 1.7 mm versus 1.2 ± 1.3 mm (*p* = 0.003) (Table 4) and days to surgery (97.0 ± 80.1 days versus 29.0 ± 253.0 days (*p* = 0.05), respectively (Table 4).

**Table 4:** Comparison of patients with or without RBH

	Patients with RBH	Patients without RBH	***p*** value*
**Number of patients**	5	421	
**Gender**			0.344
**Male**	5	319	
**Female**	0	102	
**Age**			
**Average**	31.6 ± 7.0	32.8 ± 15.3	0.856
**Range**	21 ~ 40	6 ~ 78	
**Mechanism of injury**			0.277
**Assault**	2	128	
**Traffic accident**	1	33	
**Sports**	0	87	
**Slip/Fell down**	2	85	
**Other accident**	0	88	
**Location of fracture**			0.744
**Inferior**	2	146	
**Medial**	1	154	
**Inferomedial**	2	121	
**Size of fracture**			
**Average**	11.4 ± 3.8	11.2 ± 5.2	0.703
**Range**	5.5 ~ 14.8	0.84 ~ 69.64	
**Preoperative enophthalmos**	3.6 ± 1.7	1.2 ± 1.3	0.003
**Days to surgery**			
**Average**	97.0 ± 80.1	29.0 ± 253.0	0.05
**Range**	4 ~ 163	0 ~ 5191	
**Follow-up (months)**	13.9 ± 14.9	5.1 ± 8.3	

*RBH* retrobulbar hemorrhage

* Mann-Whitney U test

When eye motility was considered as the sum of duction limitations in the 4 positions of gaze (supraduction, infraduction, adduction, and abduction), no significant intergroup difference was observed between the RBH and Control groups (− 3.1 ± 5.1 vs. -0.8 ± 1.7, respectively (*p* = 0.135)).

Five of the 426 cases involved revision surgery due to significant enophthalmos or extraocular motility restriction after previous surgery. The other 421 cases were of primary blowout fracture repair. Among 5 of the study subjects underwent revision surgery, 2 were members of the RBH group. In contrast, RBH developed in only 3 (0.7%) of the remaining 421 that underwent primary surgery. The incidence of RBH after revision surgery was significantly higher than that after primary surgery (odds ratio = 92.9 [95% confidence interval, 11.16–773.23], *p* = 0.001).

## Discussion

The possibility of permanent visual loss after ocular surgery is a serious concern to operators and patients. This study was designed to improve understanding of the prevention and treatment of retrobulbar hemorrhage by investigating the incidence and clinical characteristics of retrobulbar hemorrhage after orbital fracture repair, which is the most common type of orbital surgery.

The literature contains limited data on the incidence of RBH beyond case reports or case series. For RBH due to trauma, a retrospective review of 1386 facial trauma patients at a tertiary care trauma center emergency department reported an overall incidence of 3.6% [11]. A questionnaire-based review of 269,433 blepharoplasty cases found that the incidence of RBH was 0.055% and that of RBH with permanent visual loss was 0.0045% [6]. On the other hand, Gosau et al.in a single center series (*n* = 189) of orbital floor fracture repair reported an RBH incidence of 3.2% [7], whereas S. Lee et al. in a series of 170 patients that underwent orbital reconstruction using a porous high-density polyethylene implant found RBH was encountered in one patient (0.6%) [12]. In our 426 case series of orbital wall fracture repair performed at one academic tertiary referral center by a single surgeon, 5 patients (1.2%) developed postoperative RBH, and all 5 achieved normal visual acuity (20/20).

No specific report has been issued on the risk factors of postoperative RBH, but some have provided descriptions of prominent features in the patients. In a series of 189 orbital floor fractures, Gosau et al. reported that among 6 patients that developed RBH, three were taking anticoagulants [7]. A multisurgeon series on orbitotomy (*n* = 1665) reported that one of 4 patients that lost visual acuity due to RBH had continued aspirin immediately before and after surgery [13]. In the present study, all anticoagulant agents were stopped for a suitable period before surgery. Considering the risk of RBH, it is important to advise patients to stop any noncrucial anticoagulants before blowout fracture repair.

Extensive destruction of the orbital floor associated with complex midface fracture is another reported feature of postoperative RBH [7]. In general, extensive fractures are the result of more forceful injury mechanisms and may result in more severe cranial nerve and direct muscle injuries and more soft tissue edema [10]. But no difference in fracture size was observed between our two study groups. Since this study excluded complex facial fractures, it is thought that different results from the previous reports came out.

Regarding time between injury and surgery, despite surgical difficulties with soft tissue edema, many surgeons advocate repair of facial fractures as soon as possible after injury to optimize aesthetic outcomes [14]. Recently, Scawn RL et al. reported similar complication rates and good results for early and late orbital fracture repairs [15]. However, several reports suggest that late repairs (beyond 2 to 3 months) result in poorer outcomes [16, 17], but series were relatively small (*n* = 20, 145, 51 respectively) and no RBH event was reported. In our study, time from injury to surgery was greater in the RBH group. Despite conflicting reports on the topic, late repair of orbital fractures is technically more challenging due to scarring and adhesion of orbital soft tissues at fracture site, which may increase the risk of RBH. Generally, 3 weeks after trauma, nasal mucosa on sinus and orbital periosteum adhere to each other and are often difficult to dissect. Moreover, excessive handling often causes bleeding and requires additional manipulation to achieve hemostasis.

In this study, preoperative enophthalmos was significantly greater in the RBH group. A large amount of preoperative enophthalmos usually means a wider fracture and greater orbital volume expansion. During the early stage after injury, enophthalmos tends to be undervalued due to tissue edema and hemorrhage. However, over time, gradual fibrosis, contraction of orbital contents, and orbital fat atrophy augment enophthalmos [18–20], which cautions that attention be paid to the interpretation of preoperative results. In our study, the RBH group contained a high proportion of secondary repair patients, which probably made the preoperative enophthalmos difference appear more prominent.

No significant visual loss was noted after surgeries in the current study, but several studies have addressed visual loss after orbital surgery and reported incidences that vary widely. A single-surgeon series of 1593 orbitotomy cases for all indications reported an incidence of blindness of 0.44% [21], whereas an article on orbital cavernous hemangiomas reported a vision loss rate of 7% [22]. In recent multicenter series of 1665 orbital surgeries for all indications, 14 patients (0.84%) experienced postoperative vision loss [13], and in 4 of the 14 patients (0.24%) vision loss was the result of RBH in fracture repair cases. According to the report by Gosau et al., described above, RBH occurred in 6 of 189 subjects (3.2%) and 2 lost vision (1.06%) [7]. Another report concluded that 48% of total vision losses after orbital fracture repair were due to RBH [23].

Management of RBH starts from the prompt detection of related symptoms such as declining vision, exophthalmos, ophthalmoplegia, and pupil dilation. Diagnosis is based on clinical signs and symptoms and can be confirmed by imaging study. Considering the benefits of CT in terms of its accurately determining the location and amount of hematoma, when immediate CT is possible, it is better to undertake surgery after imaging, but if not possible, surgical treatment should not be delayed. Even a simple bed-side procedure, that is, direct intra orbital hematoma aspiration with a 23-gauge needle, can be effective for orbital decompression. In our study, one of the 5 RBH patients was salvaged by direct needle aspiration on postoperative rounding. If simple needle aspiration is ineffective, canthotomy and cantholysis, transseptal orbital incision may be performed. However, if it is determined that RBH is not severe and immediate surgical treatment is not necessary, intravenous high dose steroid or mannitol could be given under careful monitoring.

Several management methods have been suggested to prevent RBH during and after surgery. During surgery, it has been suggested that fenestrated implants such as titanium mesh, or punched alloplastic implant, may reduce the risk of RBH-related compartment syndrome by allowing bleeding from the orbit to adjacent sinuses [24–26]. In one study, it was suggested that a negative pressure drainage system using scalp vein set tube be used for RBH prevention [3]. In our experience, when excessive manipulation is applied during surgery or when there is a bleeding tendency, placement of a silastic drainage tube at the surgical site for natural drainage functions well. Conservative treatment includes avoidance of the semi-Fowler position and Valsalva manipulation, which would increase intra-orbital-pressure, and application of an icepack are also important aspects of postoperative care.

Revision blowout fracture repair is technically challenging and is one of the reasons why surgeons hesitate to operate. In the revision cases, fibrosis and adhesion from primary fracture repair can increase the risks of complications. In a study of 13 eyes that underwent secondary blowout fracture repair after unsatisfactory primary surgery, complications of pupillary obstruction in one and diplopia in three were encountered after surgery, but no case of RBH occurred [27]. In the present study, five patients underwent secondary surgery and RBH developed in two of the five, despite meticulous hemostasis and postoperative attention. This observation suggests drainage tube insertion during surgery is a good option, especially in cases of revision surgery.

Delayed orbital hemorrhage following ophthalmic surgery is rare, though a few case reports have been issued after lid surgery [4, 28, 29]. In our series, RBH timings ranged from 4 h to 12 days after surgery, which suggests it is important to hospitalize patients for several days after blowout fracture repair and to educate patients about the early symptoms of RBH. Patients and family members should be trained to test vision at home and instructed to contact a counselling team when new symptoms or concerns arise.

The limitations of this study are its retrospective, nonrandomized nature, and limited sample size. Furthermore, the size of this retrospective review prevented the use of regression analysis to identify risk factors. Nevertheless, we believe data trends justify our making recommendations.

The present study focused on the occurrence of RBH and visual acuity outcomes rather than visual field data or other measures of quality of vision. Thus, “full recovery of visual acuity” as mentioned earlier does not reflect actual damage of RBH after blowout fracture repair, as pathologic features such as visual field defects, pupillary abnormalities, and color vision deficiencies.

In summary, our findings indicate surgeons should pay more attention to hemostasis and postoperative care in patients with a large preoperative enophthalmic eye, when time from injury to surgery is long and in revision cases. Although it may not be absolutely necessary, placing of a drainage tube is worthy of consideration. When RBH occurs, time to intervention and surgical decompression is critical for visual recovery and preventing blindness. It is important that patients and doctors should know that time is the most important factor when RBH occurs, and thus, it is important to establish a system that allows effective patient-doctor communication even after discharge.

## Data Availability

The datasets analyzed during the current study are not publicly available for confidentiality reasons; nevertheless, the corresponding author will provide them on reasonable request.

## Abbreviations

RBH: Retrobulbar hemorrhage
CT: Computed tomography
